# Dynamic kinetic resolution-mediated synthesis of C-3 hydroxylated arginine derivatives

**DOI:** 10.1098/rsos.241607

**Published:** 2025-02-19

**Authors:** Ye Zheng, Zhenyu Chen, Guy J. Clarkson, Stephen A. Marshall, Jianliang Xiao, Christopher J. Schofield, Martin Wills, Andrew V. Stachulski

**Affiliations:** ^1^ Department of Chemistry, University of Warwick, Coventry CV4 7AL, UK; ^2^ Department of Chemistry, University of Liverpool, Crown Street, Liverpool L69 7ZD, UK; ^3^ Department of Chemistry, Chemistry Research Laboratory, University of Oxford, Mansfield Road, Oxford OX1 3TA, UK; ^4^ Ineos Oxford Institute for Antimicrobial Research, University of Oxford, South Parks Road, Oxford OX1 3RE, UK

**Keywords:** asymmetric synthesis, biotransformation, hydroxy amino-acids, antibiotics, catalysis

## Abstract

Hydroxylated amino acids and their derivatives, including those found in proteins, are important in biology and medicinal chemistry. Incubation of N-acetyl-l-arginine with clavaminic acid synthase, a key oxygenase in clavulanic acid biosynthesis, affords a (3R)-hydroxylated product that is identical to material obtained by total synthesis from Boc-beta alanine. The key step employed dynamic kinetic resolution (DKR) of a β-ketoester precursor, achieved in high diastereomeric and enantiomeric excess using an (R)-SEGPHOS/Ru(II) catalyst. The results highlight the utility of DKR for the preparation of C-3 hydroxylated amino acid derivatives.

## 1. Introduction

**Figure 1.**
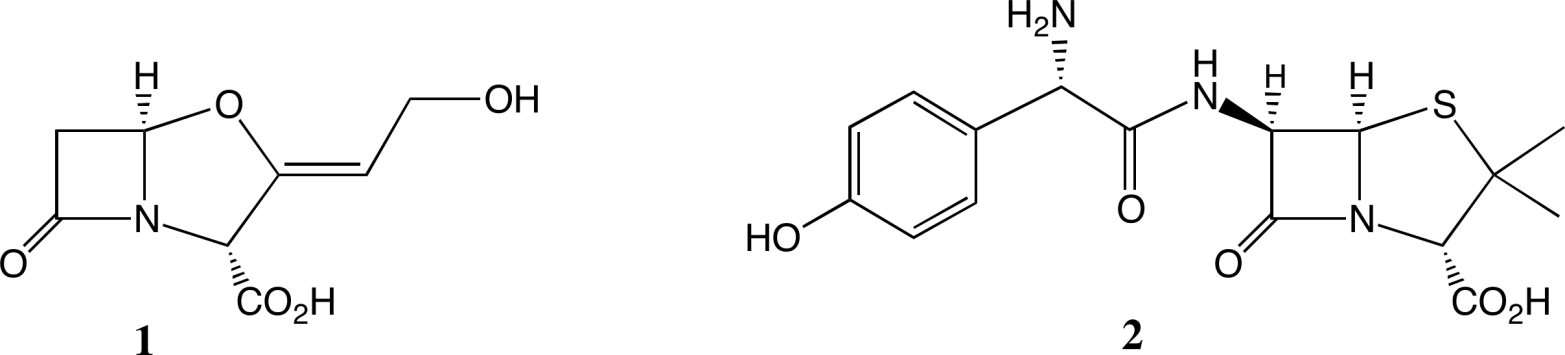
Clavulanic acid **1** and amoxicillin **2**, the constituents of augmentin.

The discovery of the *Streptomyces* metabolite clavulanic acid **1** (CA, figure 1) [1–3] was an important advance in antibacterial chemotherapy. Although **1** is a weak antibacterial *per se*, it is a potent inhibitor [4] of a range of nucleophilic serine beta-lactamases produced by bacteria as a defence against many beta-lactam antibiotics [4,5]. The combination of amoxicillin **2** with **1**, as augmentin [6] and its generic formulations, is still a vital broad-spectrum, orally absorbed antibacterial treatment. The growing threat of antimicrobial resistance, in particular via bacterial strains producing beta-lactamases [7,8], has led to a renaissance of interest in **1** and newer beta-lactamase inhibitors [9,10].

**Figure 2.**
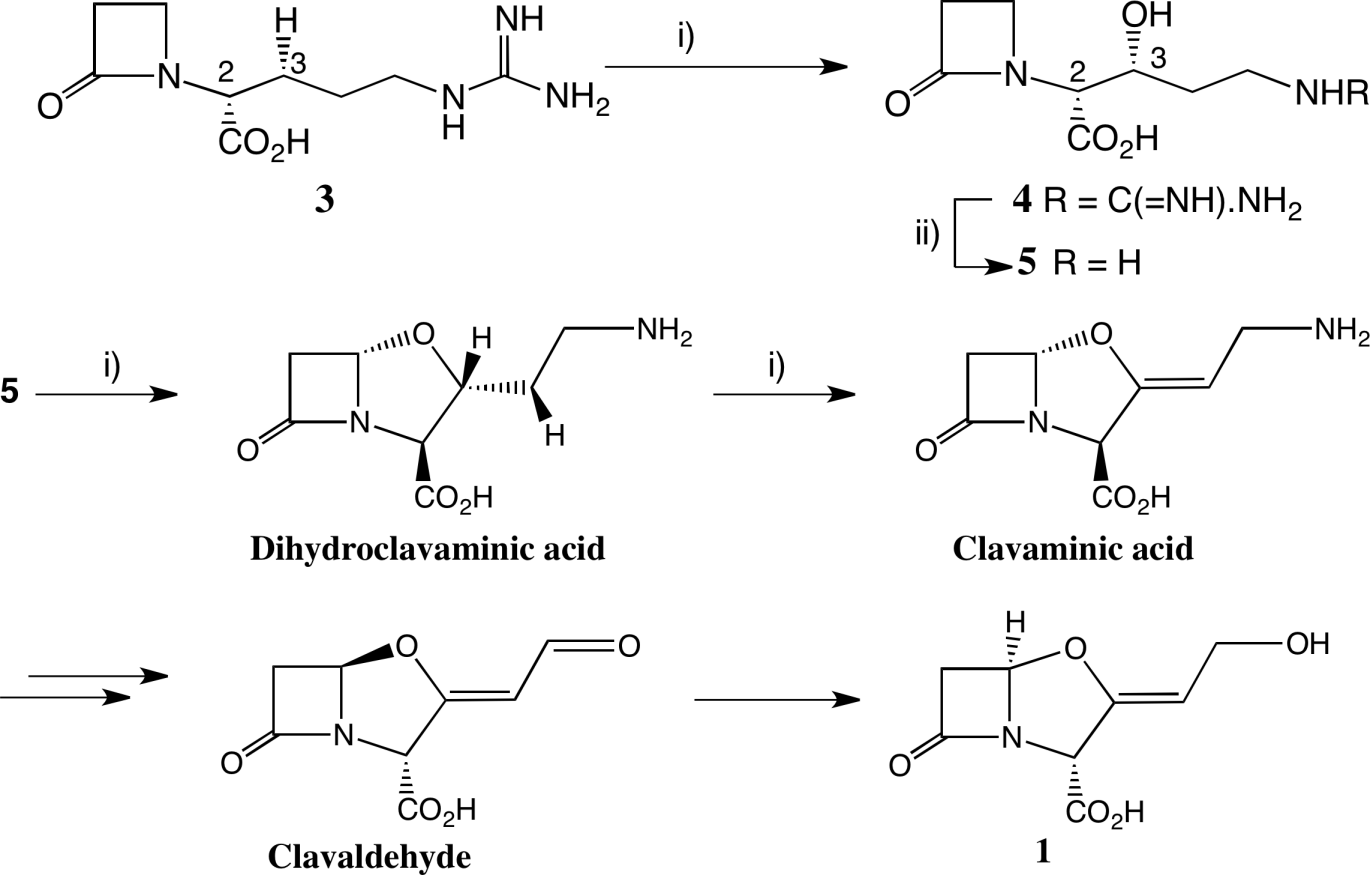
Important steps in clavulanic acid biosynthesis. (i) Clavaminate acid synthase (CAS.Fe(II)); each CAS catalysed reaction is coupled to conversion of O_2_, 2-oxoglutarate to CO_2_, succinate; (ii) proclavaminate amidino hydrolase (PAH.Mn(II)), H_2_O; urea coproduct.

There is no effective synthesis of **1**, which is commercially obtained by fermentation. An early published synthesis of *rac***-1** afforded a yield of less than 5% in a key step [11–14]. Given its importance, there has been surprisingly little structure-activity study of derivatives of CA **1**, especially of late. The biosynthesis of **1**, however, is known in some detail [14]: three early steps are catalysed by the oxygenase clavaminic acid synthase (CAS) (figure 2). A C3 hydroxyl group, the eventual source of the ring oxygen of **1**, is stereoselectively introduced into deoxyguanidino-proclavaminic acid **3** by CAS catalysis [15,16]. The guanidino group of the resultant product **4** is then hydrolysed by an amidino hydrolase (PAH) [17,18] to afford proclavaminic acid **5**, a substrate for two further CAS-catalysed reactions, that is oxidative ring closure followed by desaturation. Eventually, the final CAS product clavaminic acid is converted in further biocatalysed steps, which are not fully elucidated, to clavaldehyde, which on reduction delivers CA **1** [14].

CAS is a member of the 2-oxoglutarate (2OG)-dependent superfamily of oxygenases [19], members of which catalyse a remarkably wide range of oxidative reactions, including Lys- and Arg-residue C−3 hydroxylations. For example, Lys C−3 hydroxylation is catalysed by JMJD7 [20,21] and Arg C−3 hydroxylation is catalysed by JMJD5 [22], both of which are potential cancer drug targets [23].

**Figure 3.**
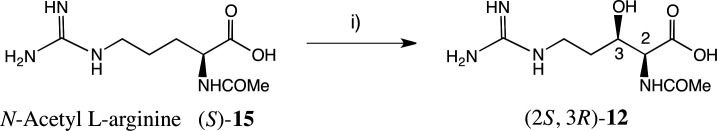
Biohydroxylation of *N*-acetyl-l-arginine (*s*)−**15**. Conditions: (i) Clavaminate acid synthase (CAS.Fe(II)); the reaction is coupled to conversion of O_2_, 2OG to CO_2_, succinate.

There is a need for efficient syntheses of C-3 hydroxylated amino acids, in order to validate structures, including stereochemistry, of biosynthetic/natural products, to act as starting points for chemo-enzymatic syntheses of drugs such as CA **1**, and to use in the generation of antigens to create antibodies selective for post-translational modifications introduced by 2OG oxygenases. In addition to the CA **1** biosynthetic intermediates, CAS also catalyses the hydroxylation of *N*-acetyl Arg to give *N*-acetyl-(2*S*,3*R*)-3-hydroxy-Arg (see figure 3), further exemplifying the biocatalytic potential of 2OG oxygenases, first demonstrated with proline hydroxylases [24–26]. However, only a single stereoisomer is produced in the CA catalysed reaction, so that its use in preparing stereoisomers is limited. Here, we report the use of dynamic kinetic resolution (DKR) for the stereoselective synthesis of both (2*S*,3*R*)- and (2*R*,3*R*)-*N*-acetylated C-3 hydroxy-Arg derivatives.

We considered that the core α-amino, β-hydroxy units of **4** and **5** could be accessible as single enantiomers via DKR [27–29] of an appropriate β-keto precursor. Indeed, the homologous 3-hydroxylysine derivatives have been prepared in this manner [30,31]. Similarly, we have shown that MeBmt, sc. (2*S*,3*R*,4*R*,6*E*)-3-hydroxy−4-methyl−2-(methylamino)−6-octenoic acid, the unusual hydroxy amino acid of cyclosporin A, can be efficiently obtained by asymmetric transfer hydrogenation (ATH) of a β-ketoanilide bearing an *N*-Me substituent, giving the desired *anti*-product [32]. Other syntheses of **5** involved a lengthy chiral pool approach [33] or a non-stereospecific aldol reaction [34].

The relative *syn*-(2*S*,3*R*)-stereochemistry in **4** and **5** could result from addition to a keto precursor under the classical Noyori hydrogenation conditions [27], namely an appropriate Ru catalyst and a high pressure of H_2_. The transfer hydrogenation mode developed by Somfai [35] and others was predicted to give *anti*-products, namely (2*S*, 3*S*)/(2*R*, 3*R*); we studied both modes. Work on a related ketoester substrate studied by Ishida, Touge *et al.* [36,37] indicated that hydrogenation using [NH_2_Me_2_][(RuCl((*S*)-Segphos®))_2_(µ-Cl)_3_] delivered the *syn* product, while an oxo-tethered derivative (DENEB) of a Noyori–Ikariya catalyst [(arene)Ru(TsDPEN)Cl] gave the *anti*-product.

**Figure 4.**
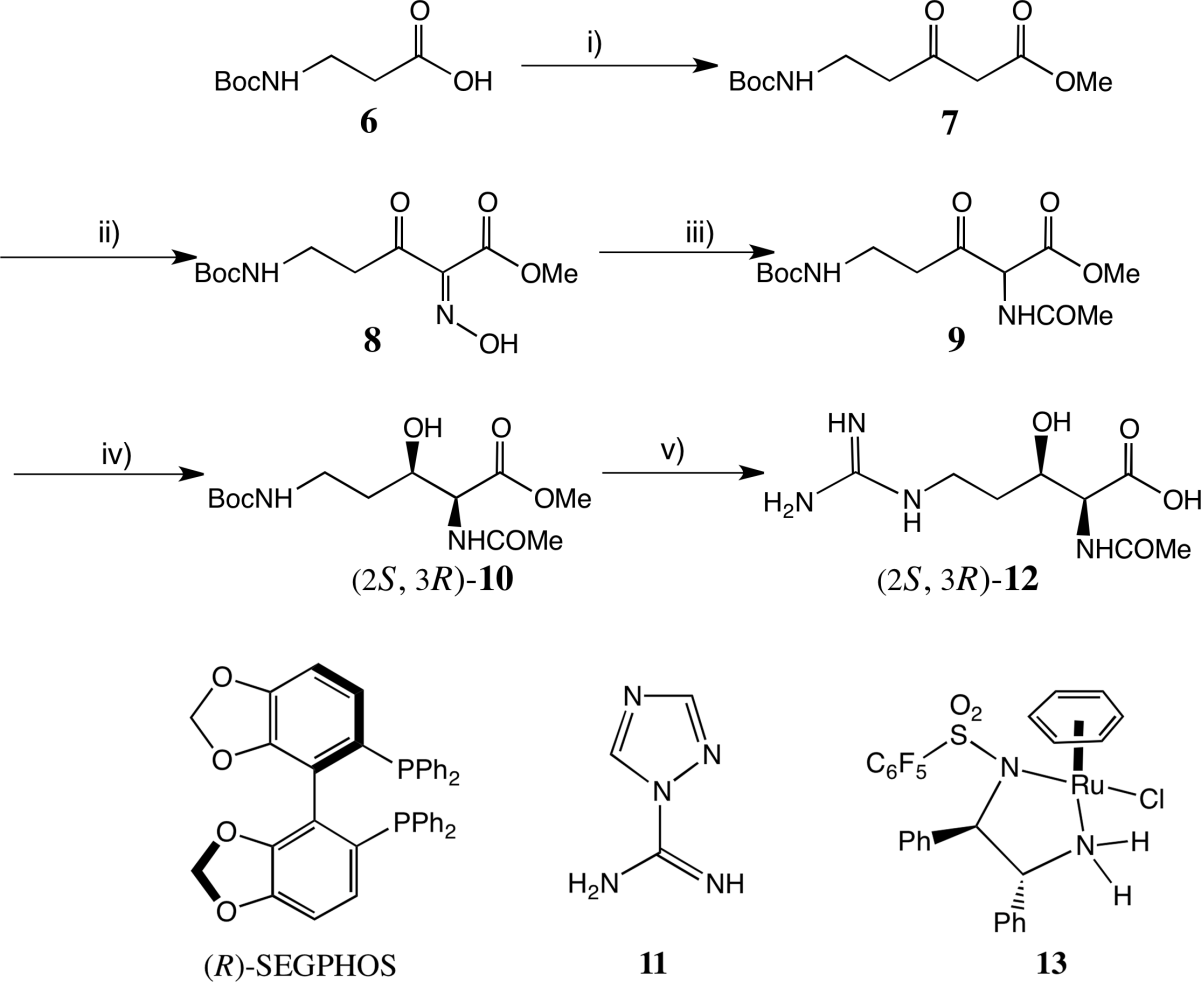
Syntheses of (2*S*,3*R*)-3-hydroxy *N*-acetyl Orn and Arg derivatives. Reagents: (i) CDI, KO_2_CCH_2_CO_2_Me, MgCl_2_, THF, 76%; (ii) NaNO_2_, AcOH, 75%; (iii) H_2_/Pd, Ac_2_O, 95%; (iv) [NH_2_Me_2_][(RuCl((R)-Segphos®))_2_(µ-Cl)_3_], CH_2_Cl_2_-MeOH, 72%; (v) (a) LiOH, aq. MeOH. (b) CF_3_CO_2_H, (c) **11**, aq. Na_2_CO_3_, purification via HPLC.

The keto precursor for all our DKR studies was efficiently obtained, as shown in figure 4 (full details are in the electronic supplementary material). Two-carbon homologation of Boc-β-alanine **6** using Masamune’s conditions using a malonate half ester, as adapted by Genet [31], gave a very good yield of β-ketoester **7** [38]. Standard nitrosation catalysed by AcOH [31] afforded the crystalline oxime **8** again in very good yield. Following the hydrogenation of the oxime, the intermediate amine was acylated with PhCOCl, affording a crystalline product, but in rather low yield. Instead, by using Ac_2_O as both solvent and reagent, the acetamide **9** resulted in an excellent yield. This proved a useful substrate for all the hydrogenations performed.

**Figure 5.**
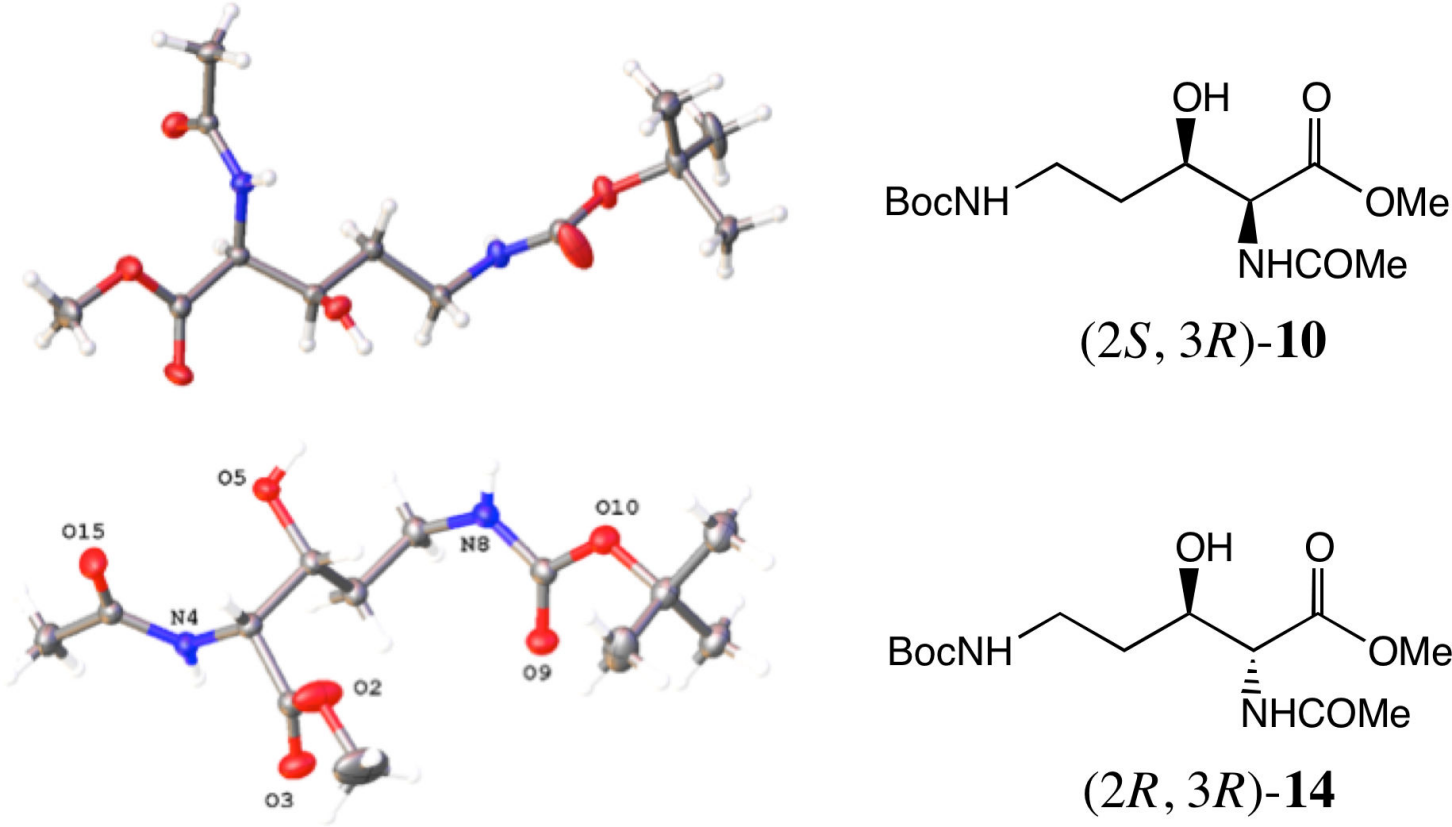
Single crystal X-ray structures of (2*S,*3*R*)-methyl 2-acetamido−5-((*tert*-butoxycarbonyl)amino)−3-hydroxypentanoate **10** and (2*R,*3*R*)-methyl 2-acetamido-5-((*tert*-butoxycarbonyl)amino)-3-hydroxypentanoate **14**.

Asymmetric hydrogenation (AH) of **9** using (*R*) or (*S*)-[NH_2_Me_2_][(RuCl(Segphos®))_2_(µ-Cl)_3_] [39,40] proved to be the method of choice for the formation of chiral *syn* products. Thus, hydrogenation of **9** at 50 atm H_2_ with (*R*)-[NH_2_Me_2_][(RuCl(Segphos®))_2_(µ-Cl)_3_] afforded (2*S,*3*R*)−**10** in 72% yield (99.6% ee, d.r. 89:11 in favour of the 2,3-*syn* product) and this was reproduced on a 1 g scale. This is a good d. r. considering the lack of branching of the C(4) methylene of **9** [35]. Recrystallization from EtOAc-hexane afforded (2*S*,3*R*)−**10** as a *single* stereoisomer whose absolute configuration was confirmed by X-ray crystallography (figure 5) [41].

To introduce the guanidino substituent present in the enzymic hydroxylation product (figure 2), the Me ester was cleaved using LiOH to minimize the risk of α-epimerization. The Boc group was cleaved from the resulting acid with CF_3_CO_2_H: the product was taken up in aq. Na_2_CO_3_ solution and treated with amidino triazole reagent **11** [42] to afford arginine derivative **12** [24–26]. This reagent appears superior to the earlier amidinopyrazole [43] and allows steady reaction at 20°C.

**Figure 6.**
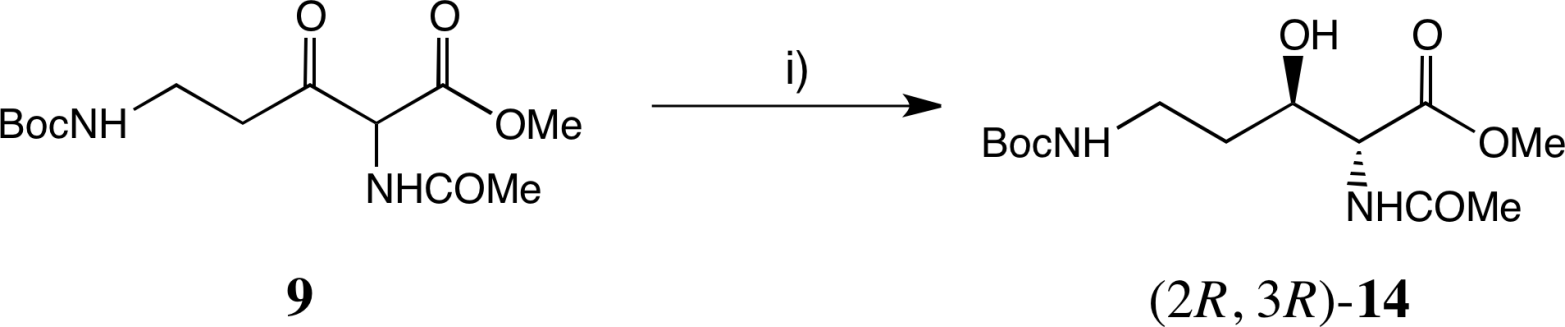
ATH of **9** using catalyst (*R*,*R*)-**13** to form (2*R*,3*R*)-**14**. Reagents: (i) 1 mol% **15**, HCO_2_H/Et_3_N (5:2), DCM, 72 h, rt, 72%.

Other hydrogenation conditions were also explored (see electronic supplementary material for full details, including chiral HPLC analyses). AH of **9** under identical conditions to the above, but using the (*S*)-SEGPHOS-based catalyst, generated (2*R,*3*S*)- or *ent*−**10** in 75% yield, 99.8% ee, d.r. 95:5. ATH of **9** was achieved using formic acid as the hydrogen donor, and a range of Noyori–Ikariya catalyst derivatives (see electronic supplementary material for full details) [44–49]. The most selective catalyst was the variant **13 [**50] with a C_6_F_5_ group on the DPEN unit (figure 4). With the catalyst containing the (R,R)-ligand, the main product was (2*R,*3*R*)-**14** (72% yield, 99% ee, d.r. 9.7:1), namely, the *anti-*diastereoisomer of **10** above (figure 6). Following recrystallization, a single stereoisomer was produced, whose structure was also confirmed by single crystal X-ray crystallography (figure 5).

The method was readily scaled up to afford gram quantities of the desired product (2*S*,3*R*)-**10**. Following conversion to the arginine derivative (2*S*,3*R*)-**12**, the material was shown by ^1^H NMR to be identical to the product of incubation of commercial *N*-acetyl-l-arginine **15** with CAS (figure 3, see electronic supplementary material for full details). It was known [25,26] that *N*-acetyl-l-arginine **15** is a superior CAS substrate to the corresponding Orn compound as it affords clean hydroxylation without the formation of an alkene byproduct.

In summary, DKR is an excellent tool for the synthesis of key C-3 hydroxylated Arg derivatives and can readily deliver gram quantities suitable for both structural/stereochemical assignment studies and for incorporation into peptides for use in antibody generation. There is clear scope for variation of the *N*α-acyl substituent, which may eventually enable a chemoenzymatic synthesis of CA **1** and its derivatives.

## Data Availability

I confirm that the data supporting this article have been included as part of the supplementary information [51].
